# The effect of deep muscle relaxation on the force required during Latissimus Dorsi dissection for breast reconstructive surgery: results of a prospective, double-blinded observational pilot study

**DOI:** 10.1186/s12871-017-0315-5

**Published:** 2017-02-21

**Authors:** T. Ledowski, A. Goodwin-Walters, P. Quinn, M. Calvert

**Affiliations:** 1 Anaesthesiology Unit in School of Medicine and Pharmacology, University of Western Australia, Level 2, Royal Perth Hospital Medical Research Foundation Building, Rear 50 Murray Street, Perth, WA 6000 Australia; 2 Department of Anaesthesia and Pain Medicine, Royal Perth Hospital, 197 Wellington Street, Perth, WA 6000 Australia; 3 Department of Plastic and Reconstructive Surgery, Royal Perth Hospital, 197 Wellington Street, Perth, WA 6000 Australia

**Keywords:** Neuromuscular blocking agents, Latissimus dorsi flap, Breast reconstructive surgery, Rocuronium, Muscle tension

## Abstract

**Background:**

The use of neuromuscular blocking agents has previously been suggested to facilitate the dissection of the latissimus dorsi muscle during breast reconstructive surgery. The aim of this study was to quantify the influence of deep muscle relaxation on the force required to lift the latissimus dorsi muscle during flap preparation.

**Methods:**

After ethics approval and written informed consent 15 patients scheduled for elective breast reconstruction with a latissimus dorsi pedicled flap (muscle flap, not myocutaneous flap) under general anaesthesia were prospectively included. Midway through the muscle dissection a sterile cotton tape was slung around the mid portion of the muscle and connected with a sterile strain gauge stably positioned just above the patient. Thereafter, the muscle was lifted by moving the strain gauge vertically upwards until a muscle tension similar to that created manually during muscle dissection was achieved. The force (N) and distance required to tension the muscle were recorded and the tension released. In a randomized and blinded crossover design either rocuronium (0.6 mg.kg^-1^) or normal saline were given intravenously, and the tension protocol was repeated 2 min after each drug administration.

**Results:**

Muscle relaxation significantly reduced the force for flap tensioning (median [percentiles] – 22 [-32/-13] %; *P* = 0.011) in 10/15 patients. However, in the remaining 5 patients no significant effect was measured. Normal saline had no effect on the measured force.

**Conclusions:**

Deep muscle relaxation significantly reduces the force required to manually elevate the latissimus dorsi muscle during its dissection in the majority of but not all patients.

**Trial registration:**

The study was retrospectively registered on [17.6.2014] with the Australian and New Zealand Clinical Trials Registry. ACTRN12614000637640

## Background

Deep neuromuscular block has recently been found to be of potential use during various forms of general surgery [1]. However, the usefulness of neuromuscular blocking agents (NMBA) for plastic and reconstructive surgery has not yet been formally investigated.

Nonetheless, after identification of the neurovascular bundle, NMBA have been suggested to facilitate the dissection of the latissimus dorsi (LD) muscle for breast reconstructive surgery [2]. Potential benefits of muscle relaxation may be the “softer” muscle requiring less manual force when raising the flap, hence facilitating dissection, as well as the absence of strong, irritating muscle twitches during electrocoagulation [3, 4].

However, the actual effect of NMBA on the tension of the LD muscle has never been investigated. Therefore, it was aim of this study to investigate the influence of deep muscle relaxation on the manual force required during muscle dissection.

## Methods

**Fig. 1:**
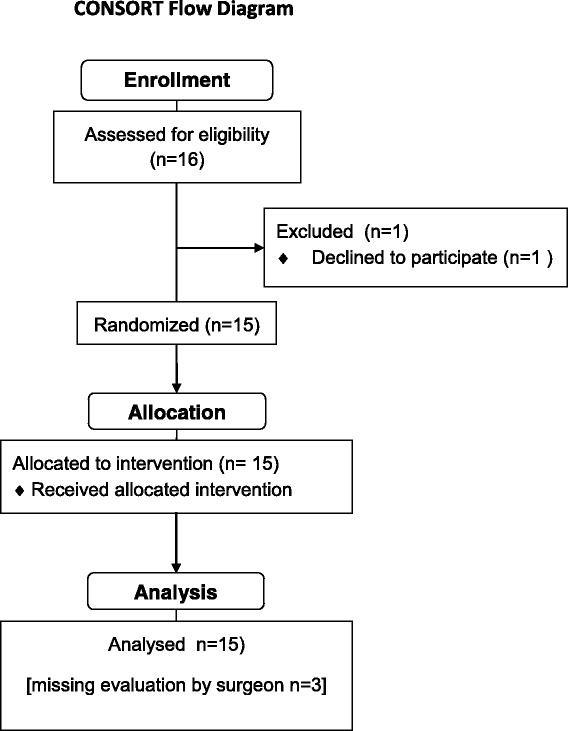
Consort flow diagram

After institutional review board approval (Royal Perth Hospital Ethics Committee EC 2012/060) patients scheduled for elective breast reconstructive surgery with a pedicled LD flap were screened for eligibility. The study was entered into the Australian and New Zealand Clinical Trials Registry (ANZCTR) under the registration number ACTRN12614000637640. A CONSORT flow chart is depicted in Fig. 1.

Included were female patients with elective LD surgery, American Society of Anesthesiology (ASA) physical status 1–3, age > 18 years, undergoing the procedure under sevoflurane/opioid/rocuronium general anaesthesia at the Royal Perth Hospital. Written informed consent was obtained from all participating patients.

Excluded were patients < 18 years, the incapacity to consent, a known allergy to the muscle relaxant rocuronium, as well as any medication or comorbidity known or suspected to interact with NMBA pharmacodynamics or the assessment thereof (i.e. myopathy, history of a stroke).

Study protocol: After induction of anaesthesia with propofol 2–3 mg.kg^-1^, fentanyl 1–3 mcg.kg^-1^ and muscle relaxation with rocuronium (maximum 0.4 mg.kg^-1^) patients were tracheally intubated. Anaesthesia was maintained with sevoflurane to achieve a standardized depth of anaesthesia (state entropy 40–60). To monitor the depth of muscle relaxation (either to confirm a state of non-paralysis or deep paralysis during the time course of the study), a kinemyometric (KMG monitor, GE Healthcare, Helsinki, Finland) device was used to measure neuromuscular transmission from the ulnar nerve to the adductor pollicis brevis muscle (supramaximal stimulation repeated every 15 s). Non-paralysis was defined as a train of four (TOF) ratio (TOFr) of > 95%, whereas deep paralysis was defined as a post tetanic count (PTC) < 5 twitches (including a profound bock with a PTC < 1).

**Fig. 2:**
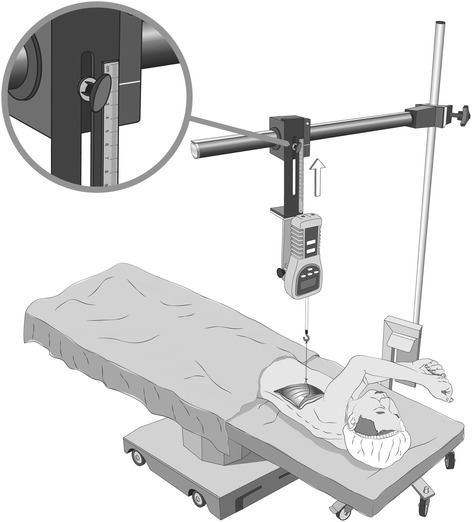
Method of force measurement with strain gauge in-situ and mid-portion of flap lifted via a sterile cotton sling. [Figure drawn for the authors by the Dept. of Medical Illustrations, Royal Perth Hospital]

The LD flap dissection was done with the patient in a lateral (*n* = 13) or prone (*n* = 2) position. After raising the mid-portion of the LD muscle leaving the origin and insertion of the muscle intact, a sterile 1 cm wide cotton tape was slung around the freed midportion of the muscle to form a loose loop. This loop was then attached via a 12 cm long sterile stainless steel rod with a terminal hook to a commercially available strain gauge (Force Gauge FG-5020; Lutron Electronic Enterprise; Taipei; Taiwan). The latter was mounted on a stable vertical stand with a horizontal arm, which was adjustable in height. This allowed positioning of the measurement instrument just above the LD muscle (see Fig. 2). At this time, non-paralysis of the patient was confirmed (TOFr > 95%; see above) and the horizontal arm of the instrument raised carefully in order to stretch and tension the muscle flap by raising it, hooked onto the cotton sling, a few centimetres off its bed just enough to mimic manual handling by the surgeon. The distance of the muscle flap elevation (mm) as well as the required force (N) were noted and the tension released thereafter. Following this “baseline” measurement, two more assessments were made in 2 min intervals. Preceding each, either normal saline (5 ml) or the muscle relaxant rocuronium (0.6 mg.kg^-1^) were given by the attending anaesthetist in random order (randomization envelope containing the order of drug administration as determined by a randomly permuted block randomization list opened by attending anaesthetist). To assess the muscular tension after either muscle relaxant or placebo (saline), the muscle was lifted by the same distance used in the baseline assessment and the force (Newton [N]) required were documented by a researcher blinded to the drug administered. Further to the assessment of forces, the (blinded to order of administration) consultant surgeon in attendance was asked to “guess” manually whether saline or muscle relaxant had been used. Three consultant surgeons were overall involved in this non-standardized (no specific scoring system used) assessment process.

At the end of the third measurement interval, all patients had received saline as well as rocuronium and were hence fully paralysed (confirmed by relaxometric assessment as described above). This marked the end of the study intervention and surgery as well as anaesthesia proceeded as clinically required. At the end of surgery, the TOF ratio was assessed and reversal (TOFr > 90%) was achieved by the administration of sugammadex (standard reversal agent for amino-steroidal NMBA in our institution), as appropriate.

### Statistics

The project was planned as a pilot investigation as to our knowledge no related data exists. Therefore no formal sample size calculation was done.

The primary outcome parameter was the force required to lift the LD flap for a pre-defined distance after either rocuronium or saline (control) administration. The secondary outcome parameter was the subjective manual assessment (patient paralysed or not) by the attending surgeon.

All data was tested for normal distribution (Shapiro-Wilk test) and are displayed as appropriate (age, body mass index (BMI) and distance of muscle lift: mean (standard deviation); all other parameters: median (25%/75% percentile; minimum and maximum value)). As the related data was found to be non-normally distributed (Shapiro-Wilk test) the Wilcoxon Signed Rank test was used to compare the forces required to lift the LD muscle.

The original data are available on request from the author.

## Results

Data of 15 patients (52 (10) years, BMI 27 (6) kg.sqm^2 -1^) were analysed. Data from all force assessments were complete. However, in 3 patients the data from the surgical assessment of paralysis was missing due to a misunderstanding during data collection.

On average, the distance of LD muscle lift was 62 (20) mm with a required force (at baseline) of median 10 N (percentiles 6.6/13.8; minimum 2.6 N; maximum 18.6 N).

In the majority of cases (*n* = 10), administration of rocuronium significantly decreased the required force (median – 22 (percentiles -32/-13; minimum -8; maximum – 44) %; *P* = 0.011).

However, in a distinctively different group of five patients (“non-responders”) the use of rocuronium did not significantly change the median force required to raise the LD muscle (median change of force after rocuronium 1 (percentiles -5/+9; minimum 0; maximum 16) %).

By touching the LD muscle, the surgeon correctly guessed whether muscle relaxant or placebo had been administered in 10/12 patients (missing data in 3 patients). This was specifically interesting in the “non-responder” (= force required unchanged after muscle relaxation) patients in whom the attending surgeon still correctly guessed when the muscle relaxant had been administered in 3/4 cases (1 incorrect guess; 5th case: missing data).

## Discussion

To the best of our knowledge this is the first study ever objectively investigating the effect of a muscle relaxant on an individual in vivo muscle relevant for plastic and reconstructive surgery. We found that the administration of a muscle relaxant significantly reduced the force likely required during manually raising the LD flap in the majority (10/15) of patients. Correspondingly, the attending surgeons subjectively felt a change (“softer muscle”) in 83% of patients after muscle relaxant administration. Not formally investigated by this trial, but empirically noted by surgeons during the investigation was the absence of strong and irritating muscle twitches when diathermy was used after the patient had been paralysed. Above described changes support the claim for a facilitating effect of muscle relaxants during LD flap surgery [2–4].

Though this generally supports the use of muscle relaxation during pedicled LD flap surgery, the described effect may not be required or even desired by every individual surgeon. As NMBA such as rocuronium have rare but potentially serious side effects, such as anaphylaxis [5], the risk-benefit ratio of using muscle relaxants during LD surgery is best discussed between surgeon and anaesthetist for each individual patient.

Surgeons in our trial recognized the effects of muscle relaxation vs saline in most patients, with an objective reduction of force by means of muscle relaxation by a median of 22%. Though to our knowledge no similar study exists, the magnitude of change is in-line with findings of studies investigating the use of muscle relaxants for laparoscopic surgery [6, 7]. After neuromuscular blockade, Lindekaer et al. [6] found an increase of abdominal workspace of approximately 9%. In a similar but larger prospective trial Dubois et al. [7] could show that muscle relaxation significantly improved surgical working conditions. It is therefore likely, that surgeons would indeed feel a difference of muscle tension in the range of 20%.

However, we were unable to measure a significant effect of muscle relaxation in 5/15 patients. Though the attending surgeon still correctly guessed whether muscle relaxant or placebo had been given in ¾ cases, we failed to measure a change of required force in the “objective”, strain gauge based, measurement. Initially, pre-operative radiotherapy treatment was suspected to have potentially caused a fibrous change in LD muscle tissue and possible scarring of the thoraco-dorsal neurovascular pedicle. However, though a post-hoc review of all patients revealed pre-operative radiotherapy in five patients, these did not turn out to be identical with the “non-responders” in the force assessment. All patients included in this study were having secondary breast reconstructions—hence the differences (i.e. scarring) between primary vs. secondary augmentation did not apply and cannot explain the group of non-responders. Furthermore, though 2 patients were operated in prone position (resulting in a different, more “natural” elevation of the LD muscle during measurements when compared to the assessment in lateral position), 1 patient was found each in the responder and non-responder group, respectively. We are hence unable to explain the non-response to muscle relaxation in a third of our patient. However, the most convincing cause may have been an inadequate mobilisation of the mid-portion of the muscle, resulting in potential inaccuracies or a large skin paddle and overlying adipose tissue bundled in the loop. As non-muscular tissue is not affected by muscle relaxation, the measurement of force changes may have been impaired. This would also explain the correct identification of muscle relaxation in all but one “non-responders” by the surgeon (surgeon touched the flap differently and may have evaluated a wider area of muscle than that assessed with the strain gauge).

This study has limitations: Firstly, only 15 patients were recruited in the pilot project. However, in this context the crossover design may add statistical power by eliminating intra-individual patients’ differences as potentially biasing factors. Secondly, though the strain gauge used was commercially available and well validated, the setup of the method of muscle tension assessment is new and has not been formerly described. In the absence of previously published similar investigations we opted to use a non-invasive technique, which was seen to best mimic the manual feeling of a surgeon during flap dissection while providing the benefit of objective force assessment.

## Conclusion

Within the limitations of a pilot study, this investigation showed a significant reduction of objectively measured force required during LD flap dissection after the use of a neuromuscular blocking agent in the majority of patients. Though this supports the use of such drugs during LD flap surgery, the use of muscle relaxants needs to be discussed between surgeon and anaesthetist in each individual case.

## Abbreviations

ANZCTR: Australian and New Zealand Clinical Trials Registry
ASA: American Society of Anesthesiology
BMI: Body Mass Index
LD: Latissimus Dorsi
N: Newton
NMBA: Neuromuscular Blocking Agent
PTC: Post Tetanic Count
TOF: Train of Four
TOFr: Train of Four Ratio
